# Supplementary material for: Sample Size and Estimation of Standard Radiation Doses for Pediatric Brain CT
Source: Tomography. 2022 Oct 1;8(5):2486–97. doi: 10.3390/tomography8050207 (PMC9612354; doi:10.3390/tomography8050207)
Supplement: Supplementary file 1 [file tomography-08-00207-s001.zip › Supplementary Materials.pdf]

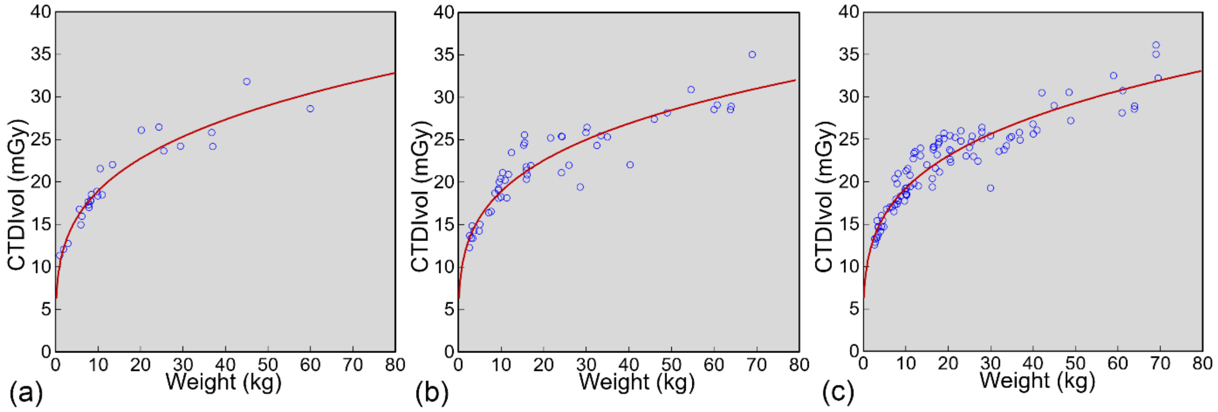

Figure S1. Examples of plots of CTDIvol against weight for small (**a**), medium (**b**), and large (c) datasets. Red curves indicate power functions fitted to the plots.

Table S1. Coefficients of determination between CTDIvol and age.

Fitting method	S	M	L
Logarithmic	0.875 (0.683–0.936)	0.875 (0.833–0.922)	0.867 (0.830–0.908)
Power	0.903 (0.676–0.956)	0.897 (0.864–0.939)	0.896 (0.856–0.932)
Bilinear (young)	0.904 (0.624–0.960)	0.883 (0.797–0.964)	0.886 (0.806–0.961)
Bilinear (old)	0.777 (0.465–0.910)	0.751 (0.651–0.902)	0.761 (0.661–0.830)

Median values are presented with ranges in parenthesis.

Table S2. Coefficients of determination between CTDIvol and weight.

Fitting method	S	M	L
Logarithmic	0.948 (0.724–0.973)	0.945 (0.917–0.971)	0.942 (0.914–0.962)
Power	0.945 (0.710–0.973)	0.940 (0.896–0.973)	0.940 (0.911–0.960)
Bilinear (light)	0.930 (0.793–0.984)	0.948 (0.840–0.971)	0.923 (0.881–0.958)
Bilinear (heavy)	0.803 (0.497–0.920)	0.794 (0.575–0.899)	0.781 (0.642–0.867)
